# Optimized Ensiling Conditions and Microbial Community in Mulberry Leaves Silage With Inoculants

**DOI:** 10.3389/fmicb.2022.813363

**Published:** 2022-06-02

**Authors:** Xiaopeng Cui, Yuxin Yang, Minjuan Zhang, Feng Jiao, Tiantian Gan, Ziwei Lin, Yanzhen Huang, Hexin Wang, Shuang Liu, Lijun Bao, Chao Su, Yonghua Qian

**Affiliations:** College of Animal Science and Technology, Northwest A&F University, Yangling, China

**Keywords:** mulberry leaves, inoculant, ensiling conditions, ensiling characteristics, microbial community

## Abstract

Mulberry leaves (ML) are a promising alternative fodder source due to their high protein content and the abundance of active components. A test of three inoculants in various combinations revealed that high-quality ML silage was produced at an inoculum ratio of 1:1:0 (50% *Saccharomyces cerevisiae*, 50% *Lactobacillus plantarum*, and 0% *Bacillus subtilis*). Using dry matter (DM) loss, pH, ammonia-N and amino acid contents, total antioxidant activity, and total flavonoids content to evaluate silage quality, this inoculant mixture was shown to produce high-quality silage within a range of inoculum size (5–15%), moisture contents (50–67%), ensiling temperatures (27–30°C), and ensiling duration (14–30 days). A third trial comparing silages produced after 30 days at 28°C and 50% moisture content revealed that silage E, prepared using an *L. plantarum* inoculant alone, displayed the lowest DM loss and pH, and low bacterial diversity, and it was dominated by *Lactobacillus* (88.6%), with low abundance of *Enterobacter* (6.17%). In contrast, silage B5, prepared with equal ratios of *L. plantarum* and *S. cerevisiae*, was dominated by *Enterococcus* (67.16%) and *Lactobacillus* (26.94%), with less marked yeast persistence, and reducing the DM content from 50 to 40% altered these relative abundances to 5.47 and 60.61, respectively. Control silages produced without an inoculant had the highest pH and ammonia-N content (indicative of poor quality), had the lowest antioxidant activity, had higher bacterial diversity, and were dominated by *Carnobacterium* (74.28%) and *Enterococcus* (17.3%). In summary, ensiling of ML conditions with proper inoculants yielded high-quality silage with a favorable microbial community composition.

## Graphical Abstract

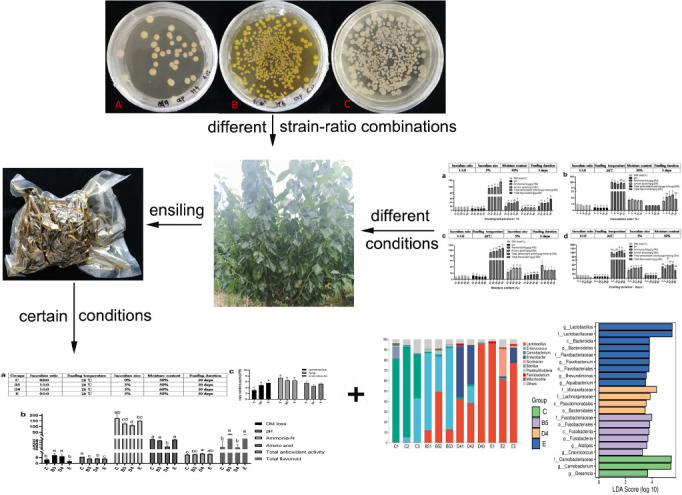

## Introduction

Mulberry (*Morus alba*), which originated in China and Korea, has been cultivated in China for more than four millennia. Mulberry leaves (ML) are known for their wide adaptability (Sugiyama et al., 2016), palatability, high protein content [18–25% in dry matter (DM)], and high *in vivo* DM digestibility (75–85%; Kandylis et al., 2009), with the absence of or negligible anti-nutritional factors (Srivastava et al., 2006). In addition, as one of the Chinese traditional medicines and edible foods, ML contain a variety of components with biological activity, including 1-deoxynojirimycin (DNJ), polysaccharides, flavonoids, and polyphenols. These biologically active components have reported anti-obesity, anti-hyperglycemic, and anti-inflammatory effects (Jeszka-Skowron et al., 2014; Ren et al., 2015). Accordingly, ML can be considered as an alternative “green” additive replacing the use of antibiotics to promote animal growth and improve the endurance and disease resistance of livestock (Zhao et al., 2015). In addition, ML are widely used in food and tea preparations in China, so they are safe for animals and human beings.

Ensiling is a traditional and effective method for the preservation of fresh forage, which can improve palatability and nutrient content and provide good nutrition for livestock throughout the year. With the gradual application of additives to improve the quality of silage fermentation, the use of bacterial inoculants, such as yeast, *Lactobacillus*, *Bacillus*, and, in particular, *Lactobacillus plantarum*, has become increasingly common practice in silage production (Yan et al., 2019; Zhao et al., 2020). Furthermore, considering that silage is the product of microbial fermentation, silage quality is likely to be strongly influenced by inoculum size and environmental factors, including temperature and moisture content. Moreover, the duration of ensiling is also closely related to the abundance of bacteria in silage (Wang et al., 2020).

As a kind of untraditional feed, ML exhibit several advantages over other forages. Hence, it is vital to explore the appropriate ensiling conditions in order to control the quality and the composition of the bacterial community of ML silage. Such knowledge is crucial to better understand the ensiling process of ML and to support its wider adoption. Therefore, in this study, optimal conditions for ML ensiling were determined by assessing fermentation characteristics and changes in the bacterial community during ensiling. The results presented in this study provide a theoretical basis and technical support for the practical implementation of ML ensiling.

## Materials and Methods

### Inoculant Activation

Strains of *Saccharomyces cerevisiae* (*S. cerevisiae*, CICC 31105), *L. plantarum* (CICC 23941), and *Bacillus subtilis* (*B. subtilis*, CICC 10012) were purchased from the China Center of Industrial Culture Collection (www.china-cicc.org) and were activated referring to the manufacturer’s protocols. *S. cerevisiae*, *L. plantarum*, and *B. subtilis* were grown on potato dextrose agar (PDA, Beijing Aoboxing Biotechnology Co., Ltd., Beijing, China) at 30°C for 24 h, de Man, Rogosa and Sharpe agar (MRS, Beijing Land Bridge Technology Co., Ltd., Beijing, China) at 37°C for 24 h, and Luria-Bertani (LB, consists of 1% peptone, 0.5% yeast extract, 1% sodium chloride, and 1.5–2% agar) at 37°C for 24 h, respectively. Colony morphology of the three strains is shown in Supplementary Figure 1. Then, three strains were resuspended and grown in a solution of nutrient broth for 3 days at 37°C prior to application in subsequent ensiling experiments.

### Silage Preparation and Experimental Design

Mulberry leaves were harvested in May 2020 at the Institute of Sericulture and Silk in Wuquan, Shaanxi Province, China. Then, they were treated with the following variables: various inoculant ratios (1:1:0, 1:0:1, 0:1:1, 1:1:1, 2:1:1, 1:2:1, 1:1:2, 2:2:1, 2:1:2, 1:2:2, and 0:0:0 exhibited by ratios of *S. cerevisiae*, *L. plantarum*, and *B. subtilis*; trial 1); various inoculum sizes (1, 5, 8, 10, and 15%); various fermentation temperatures (12, 27, 30, 37, and 50°C); various moisture contents (33, 40, 50, 60, and 67%); various ensiling duration (3, 7, 14, 21, 30, and 42 days; trial 2), which were conceived with a single-factor experimental design to determine superior conditions for ML ensiling. Subsequently, trial 3 was conducted using chosen conditions based on trials 1 and 2 to optimize ensiling conditions applied to practical promotion. Detailed experimental design is shown in the section “Results and Discussion”.

After complete homogenization, each treatment mixture (approximately 30 g) was vacuum sealed in vacuum bags made with food-grade PA/PE composite material (approximately 10 × 25 cm, 16 cm thickness, No. 14914, purchased from Taobao at https://m.tb.cn/h.fG0hNxi?tk=N0lj2kuFnJQ) to enable fermentation. Three bags of each treatment were sampled to determine the dynamic profiles of ensiling characteristics and bacterial community composition. Notably, the moisture content of ML silages was controlled by the addition of sterile water to sun-dried ML, considering the limited availability of fresh ML, whose harvesting occurs from May to August every year.

### Analysis of Microbial Population, Organic Acid Content, and Chemical Composition of Mulberry Leaves Silage

According to Liu et al. (2019), 5 g of the sample was immediately blended with 45 ml of sterilized saline water (0.85% NaCl), and the mixture was serially diluted from 10^–1^ to 10^–6^. Colonies of lactic acid bacteria (LAB) were enumerated, as viable numbers of microorganisms in the samples were expressed as the colony forming unit (CFU) per gram of fresh matter on MRS agar. Five grams of each silage sample was mixed with 45 ml of distilled water, stored at 4°C for 18 h, and then filtered. The pH of this filtrate was measured using a glass electrode pH meter (PHS-25; Shanghai Ridao Co., Ltd., Shanghai, China). Crude protein content was determined using the Kjeldahl method in a Kjeltec™ 8400 Auto-Analyzer (FOSS Analytical Instruments Inc., Hillerød, Denmark).

Fresh silage was dried on oven drying (DGX-9073BC-1) at 65°C for 48 h to obtain dry weight, and dry matter loss (DM loss) of ML silage was calculated by the following formula: DM loss = (fresh weight – dry weight)/dry weight. The content of ammonia-N was determined following the method by Broderick and Kang (1980). The content of water-soluble carbohydrates (WSC) was determined by referring to the anthrone method (Murphy, 2010). Contents of amino acids and alkaloids and total antioxidant capacity (DPPH) were determined using commercially available kits (AA-2-W, SWJ-2-Y, DPPH-2-D; Suzhou Keming Biological Co., Ltd.) according to the manufacturer’s instructions. The content of organic acids [lactic acid (LA) and acetic acid (AA)] was measured using high-performance liquid chromatography (HPLC) [Column Kromasil C_18_ (250 mm × 4.6 mm; 5 μm); oven temperature: 30°C; mobile phase: sodium phosphate buffer solution (12.5 mM); pH: 4.0; flow rate: 1.0 ml/min; injection volume: 5 μl; using an ultraviolet detector]. Contents of total flavonoids, polysaccharides, and polyphenols were measured by ethanol extraction, phenol-sulfuric method, and the Folin-Ciocalteu method, respectively. The content of 1-deoxynojirimycin (DNJ) was determined by the enzyme-linked immune-sorbent assay using a commercial kit (ZT-20586; Fankew, Shanghai FANKEL Industrial Co., Ltd.).

### Isolation of Microbial DNA and Real-Time Fluorescence Quantitative PCR for Microbial Composition

The DNA extraction was performed according to the method proposed by Liu et al. (2019). Three sample bags of each treatment (ensiling temperature and duration of trial 2 and trial 3) were taken for the analysis of microbial composition by real-time fluorescence quantitative PCR (qPCR). Approximately 10 g of the sample was mixed with 90 ml of sterile 0.85% NaCl under vigorous shaking at 120 rpm for 2 h. The mixture was filtered through four layers of cheesecloth, and the filtrate was centrifuged at 10,621 × *g* at 4°C for 10 min. The precipitate was resuspended in 1 ml of sterile 0.85% NaCl, and the microbial pellets were obtained by centrifugation at 15,294 × *g* at 4°C for 10 min. Total DNA was extracted using the OMEGA stool DNA Kit (D4015; Omega Bio-tek Inc., Norcross, GA, United States) according to the manufacturer’s instructions.

Primers used in real-time qPCR performed in this study were designed for *Lactobacillus* (UF-lac: TTTAYGCGGAACAYYTR GGKGT, UR-lac: CCAAACATCACVCCRACTT), fungi (F: TGACTCAACACGGGGAAACT, R: CCAACTAAGAACGGC CATGC), and *Enterobacter* (F: ATCAGATGTGCCCAG ATGG, R: CCGTGTCTCAGTTCCAGTG) and were synthesized by Sangon Biotech (Shanghai) Co., Ltd. (Shanghai, China). Sample DNA and plasmid DNA were serially ten-fold diluted from 10^8^ to 10^0^ copies/μl and then employed in the real-time qPCR assay. To draw standard curves for absolute quantification of the three bacteria in the samples, low copy number samples were analyzed only when the coefficient of the standard curve regression was higher than 0.98 and the run efficiency was higher than 90%. PCR was performed using a 20-μl reaction mixture containing 10 μl of 2 × SYBR Green *Pro Taq* HS Premix, 0.4 μl of each primer (5 μM), 2 μl of template DNA, and 7.2 μl of RNase free water and was guided by 30 s of denaturation at 95°C, 40 cycles of 5 s at 95°C, 30 s for annealing at 60°C, followed with solubility curve (default setting) using Roche Light Cycler^®^96. For every repetition of each sample, PCR amplifications were conducted in triplicate. The number of copies of the rRNA gene was expressed as log_10_ copy numbers/g of fresh matter (FM).

### Analysis of Microbial Community

Microbial 16S rRNA gene sequencing and bioinformatic analysis were conducted by Shanghai Personal Biotechnology Co., Ltd. (Shanghai, China). Total genomic DNA was extracted using the OMEGA Soil DNA Kit (D5625-01; Omega Bio-tek Inc.) following the manufacturer’s instructions and stored at –20°C until further analysis. Quantity and quality of extracted DNAs were measured using a NanoDrop ND-1000 spectrophotometer (Thermo Fisher Scientific, Waltham, MA, United States) and agarose gel electrophoresis, respectively. PCR amplifications of the V3–V4 region of the bacterial 16S rRNA gene were performed using the forward primer 338F (5′-ACTCCTACGGGAGGCAGCA-3′) and the reverse primer 806R (5′-GGACTACHVGGGTWTCTAAT-3′). Sample-specific 7-bp barcodes were incorporated into the primers for multiplex sequencing. The PCR components contained 5 μl of buffer (5 ×), 0.25 μl of Fast pfu DNA polymerase (5U/μl), 2 μl (2.5 mM) of dNTPs, 1 μl (10 μM) of each forward and reverse primer, 1 μl of DNA template, and 14.75 μl of ddH_2_O. Thermal cycling consisted of initial denaturation at 98°C for 5 min, followed by 25 cycles consisting of denaturation at 98°C for 30 s, annealing at 53°C for 30 s, and extension at 72°C for 45 s, with a final extension of 5 min at 72°C. PCR amplicons were purified with Vazyme VAHTSTM DNA Clean Beads (Vazyme, Nanjing, China) and quantified using the Quant-iT PicoGreen dsDNA Assay Kit (Invitrogen, Carlsbad, CA, United States). After the individual quantification step, amplicons were pooled in equal amounts, and pair-end 2 × 250 bp sequencing was performed using the Illumina MiSeq platform according to Wang et al. (2018) with slight modification. Noisy sequences of raw tags were filtered using the QIIME 2 (2019.4) under specific filtering conditions to obtain high-quality clean tags. Sequences that contained > 10% of unknown nucleotides (N) and < 80% of bases with quality (Q-value) > 20 were removed. Paired-end clean reads were merged as raw tags using FLSAH (v1.2.7) with a minimum overlap of 10 bp and mismatch error rates of 2%. The effective tags were clustered into operational taxonomic units (OTUs) at 97% similarity using QIIME 2 dada 2. Bioinformatic analysis was performed with QIIME2 v. 2019.4 (https://docs.qiime2.org/2019.4/tutorials/) and the website at https://www.genescloud.cn. Analysis of sequencing data was mainly performed using QIIME2 and R packages (v3.2.0). The sequence data have been deposited in the NCBI database (Accession No. PRJNA782016).

### Statistical Analysis

Statistical analysis was performed by the SPSS 19.0 software (IBM-SPSS Statistics, IBM Corp., Armonk, NY, United States). Data were evaluated using a one-way ANOVA followed by Fisher’s multiple range tests for ensiling quality parameters and microbial counts. Significance was declared if *P* < 0.05. Additionally, data of high-throughput sequencing were analyzed using Genescloud tools, a free online platform for genomic data analysis (https://www.genescloud.cn/chart/).

## Results and Discussion

### Ensiling Characteristics of Mulberry Leaves Silage Obtained With Different Inoculant Ratios (Trial 1)

#### Characteristics of Pre-ensiled Mulberry Leaves

Chemical composition and diversity of the microbial population of pre-ensiled ML are shown in Table 1. Contents of crude protein, crude fiber, and amino acid were 19.13%, 27.60%, and 10.40% DM, respectively, which were comparable with the contents in ML found by Wang et al. (2019b). ML could be an alternative supplemental feed with substantial crude protein, comparable to alfalfa, to replace conventional feed (Wang et al., 2012). According to Cai et al. (1998), a WSC content greater than 5% DM is necessary for desirable silage quality, especially for an inoculant fermentation (Muck, 2010). In this regard, the WSC content in ML (4.59% DM), although higher than that in forage soybean (1.23% DM) and lower than that in grass forage (crop corn 13.69% DM; sorghum 12.23% DM; Ni et al. (2018), was slightly sufficient for the extent and rate of an adequate fermentation and indicated some DM loss during fermentation. However, the DM content of ML (38.09%) was far higher than the minimum DM content (25%) indicated for forage to minimize the risk of effluent of nutrients (Mcdonald et al., 1991) and presumably alleviated DM loss during ensiling.

**TABLE 1:** Chemical and microbial composition of pre-ensiled mulberry leaves.

Characteristics	Contents
DM (%)	38.09 ± 3.80
Crude protein (%DM)	19.13 ± 1.09
Crude fiber (%DM)	27.60
Amino acid (mg/g DM)	10.40 ± 0.16
WSC (mg/g DM)	45.87 ± 2.17
Total flavonoid (mg/g DM)	20.97 ± 0.44
Polysaccharide (mg/g DM)	9.48 ± 0.19
Total polyphenol (mg/g DM)	2.67 ± 0.05
Alkaloids (μg/g DM)	566.26 ± 57.63
DNJ (× 10^–4^ ng/g DM)	9.21 ± 0.37
*Lactobacillus* (log_10_CFU/g FM)	2.12 ± 0.74
Fungi (log CFU/g FM)	2.81 ± 0.04
*Enterobacter* (log CFU/g FM)	3.09 ± 0.03

*DM, dry matter; FM, fresh material.*

Contents of total flavonoids, polysaccharides, total polyphenols, alkaloids, and DNJ were 20.97 mg/g DM, 9.48 mg/g DM, 2.67 mg/g DM, 566.26 μg/g DM, and 9.21 μg Trolox/g DM, respectively. It is widely known that these active ingredients are beneficial to the growth and health of animals (Li et al., 2019).

In addition, the microbial load of ML was determined using real-time qPCR. Overall, the load in log_10_ CFU/g of *Lactobacillus* (2.12) was lower than that of fungi (2.81) and *Enterobacter* (3.09). Moreover, the load of *Lactobacillus* in ML in this study, as the dominant bacterial species for an exceptional fermentation, was not only below the minimum adequate number (< 10^5^ CFU/g) known to effectively improve silage quality but also under 10^4^ CFU/g, which might have contributed to reduce DM recovery and increase ammonia concentration (Oliveira et al., 2017). Accordingly, the addition of inoculants to modulate bacterial community composition in ML is strongly advisable.

#### Sensory Analysis of Mulberry Leaves Silage

After ensiling at 30°C for 3 days with 5% inoculum size and 50% moisture content of ML, smell, color, and shatter value of all treatment groups changed according to the addition of different inoculant ratios compared with the control group (W), which showed less gas production, putrefactive odor, slight caking, and blue green color, all indicative of further decay of ML silage (Table 2). Similarly, ML silages prepared with inoculant ratios of 1:0:1 and 1:2:2 had better quality than W, since they showed less gas production, faint foul odor, and a slimy feel, which can be attributed to the activity of bacilli (Muck, 2010). Other treated groups had similar sensory evaluation profiles: low gas production, acid fragrance, loose, and faint yellow color. Collectively, when the existing population of *S. cerevisiae* and *B. subtilis* ≥ 60% and *S. cerevisiae* < *B. subtilis* or *L. plantarum* was absent in the inoculants, it would result in ML silage with a faint foul odor caused by spoilage bacteria with prolonged ensiling. The reasons seemed that *B. subtilis* caused the increase in pH (perhaps assisted by yeasts in some instances; Barry et al., 1980) and indole production from degradation of tryptophan, thereby accelerating spoilage of ML silage.

**TABLE 2:** Evaluation of sensory profile of mulberry leaves silage obtained with different inoculant ratios presented by *S. cerevisiae*, *L. plantarum*, and *B. subtilis* in **trial 1**.

Inoculant ratio	Gas production	Smell	Color	Shatter value
1:1:0	Remarkable	Acid fragrance	Faint yellow	Loose
1:0:1	Less	Faint foul odor	Blue green	Loose
0:1:1	Remarkable	Acid fragrance	Faint yellow	Loose
1:1:1	Remarkable	Acid fragrance	Faint yellow	Loose
2:1:1	Remarkable	Acid fragrance	Faint yellow	Loose
1:2:1	Remarkable	Acid fragrance	Faint yellow	Loose
1:1:2	Remarkable	Acid fragrance	Faint yellow	Loose
2:2:1	Remarkable	Acid fragrance	Faint yellow	Loose
2:1:2	Remarkable	Acid fragrance	Faint yellow	Loose
1:2:2	Less	Faint foul odor	Faint yellow	Loose
W(0:0:0)	Less	Putrefactive odor	Blue green	Slight caking

#### Ensiling Characteristics of Mulberry Leaves Silage

As presented in Table 3, ensiling characteristics of treated ML silage related to ammonia, pH, organic acids content, and microbial load changed greatly compared with W. DM loss mainly derived from aerobic activity at the early stage of ensiling, likely as a result of the metabolism by aerobic microorganism (e.g., yeast and bacillus) and volatile production during heterofermentation, which enabled the conversion of WSC to ethanol (Muck, 2010; Avila et al., 2014), thus resulting in DM loss (Oladosu et al., 2016) while simultaneously improving palatability. After the addition of inoculants at different ratios, an indistinctive increase in DM loss without 0:1:1 was observed compared with W, probably due to the antibacterial effect of ML on microorganisms during fermentation. Also, more DM loss was observed from *Bacillus* inoculants than from yeast inoculants. As expected, compared with W, a significant decrease in WSC, as well as in crude protein and amino acid content, was observed in treated ML silages except for treatment 1:0:1. The excessive crude protein content in ML silage obtained with the inoculant ratio of 1:0:1 might be attributed to fungal activity, since Zhao et al. (2020) reported that the crude protein content of corn straw in all fungal treatments increased, peaking on day 35.

**TABLE 3:** Ensiling parameters and chemical contents of mulberry leaves ensiled with the addition of different inoculant ratios in **trial 1**.

Treatments	DM_*loss*_	Ammonia-N	pH	LA	AA	LAB	Crude protein	Amino acid	WSC

	%	μg/g FM		mg/g FM	mg/g FM	log_10_CFU/g FM	%DM	mg/g DM	mg/g DM
1:1:0	4.53^ab^	99.27^de^	4.22^b^	83.91^a^	76.67^a^	6.78^a^	22.92^b^	20.93^b^	16.16^b^
1:0:1	4.12^ab^	119.47^a^	4.99^a^	51.97^e^	52.42^d^	5.38^c^	27.85^a^	23.69^a^	42.60^a^
0:1:1	5.06^a^	93.70^e^	4.26^b^	70.60^b^	65.68^b^	4.89^d^	22.98^b^	21.87^ab^	13.90^b^
1:1:1	3.97^ab^	105.93^cd^	4.24^b^	65.69^c^	61.31^c^	5.87^b^	23.12^b^	23.11^a^	15.53^b^
2:1:1	3.18^ab^	106.04^cd^	4.21^b^	56.03^d^	51.40^de^	4.83^d^	23.21^b^	22.49^ab^	15.57^b^
1:2:1	4.28^ab^	107.96^abcd^	4.23^b^	41.48^f^	38.19^f^	4.81^d^	20.66^bc^	22.01^ab^	16.72^b^
1:1:2	3.56^ab^	109.17^abcd^	4.25^b^	39.69^f^	37.58^f^	4.20^fg^	19.13^c^	22.12^ab^	16.04^b^
2:2:1	3.02^ab^	107.54^bcd^	4.24^b^	54.14^de^	48.18^e^	4.36^f^	23.05^b^	21.49^ab^	15.23^b^
2:1:2	3.16^ab^	107.33^bcd^	4.23^b^	53.85^de^	52.01^d^	4.59^e^	23.13^b^	20.59^b^	15.83^b^
1:2:2	3.12^ab^	114.98^abc^	4.22^b^	56.25^d^	49.86^de^	4.10^g^	23.21^b^	21.07^b^	16.49^b^
W	1.93^b^	118.92^ab^	4.74^a^	42.16^f^	51.18^de^	4.01^g^	22.72^b^	22.56^ab^	42.36^a^
SEM	0.267	1.630	0.050	2.271	1.932	0.142	0.422	0.248	1.884
*P*-value	0.582	0.005	0.000	0.000	0.000	0.000	0.001	0.189	0.000

*SEM, standard error mean. Different letters in the same column (a–g) differed (P < 0.05).*

Silage pH plays a vital role in silage quality. pH 4.2 is commonly considered as a well-fermented benchmark, particularly for high moisture silage, and a lower pH generally ensures adequate fermentation, good aerobic stability, and long preservation. However, the exact pH to inhibit these groups does vary by crop and DM content in addition to the bacterial strains present (Muck, 2010). In this study, compared with W (4.74), pH rapidly declined in ML silage (*P* < 0.05) to a relatively low value (∼4.2) at the early stages of ensiling, except for 4.99, an unfavorable pH to silages in treatment 1:0:1 due to the absence of *L. plantarum*. A similar finding (pH 4.31–4.69 for treatments compared with pH 6.07 for the control) was obtained when investigating the role of the microbiota in the fermentation of oat silage (Wang et al., 2020). In general, the pH decline is initiated by the generation of organic acids during ensiling and is greatly influenced by the acid concentration and buffering capacity of materials (Kung et al., 2018). However, the final pH of silage is also dependent on many factors (e.g., ammonia-N content; Barry et al., 1980; Table 2 and Supplementary Table A3).

Lactic acid (p*K*_*a*_ of 3.86) is generally produced from the conversion of WSC mainly by homofermentative LAB, and it greatly contributes to a rapid decrease in the pH of silage, due to the fact that it is approximately 10–12 times stronger than other major organic acids [e.g., acetic acid (p*K*_*a*_ of 4.75) and propionic acid (p*K*_*a*_ of 4.87)] (Kung et al., 2018). Additionally, acetic acid dramatically inhibits yeast and mold growth produced by heterofermentative LAB, such as *Lactobacillus buchneri*, with a remarkable effect on improving the aerobic stability at feeding (Muck, 2010; Kung et al., 2018). As expected, treatment 1:1:0 indicated a well-preserved silage with the highest LA, AA, and LAB (Table 3). Moreover, both ≥ 50% of *L. plantarum* and the absence of *B. subtilis* were indispensable to lift the levels of LA and AA.

Ammonia-N is a common indicator of protein degradation, reflecting peptide bond hydrolysis and a higher rate of amino acid and/or peptide deamination (Li et al., 2018). In this study, over 105 μg/g FM of ammonia-N was produced in all treatments, except in treatments 1:1:0 (99.27 μg/g FM) and 0:1:1 (93.70 μg/g FM). This point might be clarified by the unfavorable role of *S. cerevisiae* and *B. subtilis* (≥50% of total addition) to silage, where bacillus has strong proteolytic activity and can decompose protein and produce ammonia. Meanwhile, the main fermentation products of yeasts are ethanol and CO_2_ due to the metabolism of lactic acid (Pahlow et al., 2003; Muck, 2010). Therefore, *S. cerevisiae* and *B. subtilis* should not be applied simultaneously to ML silage, and a minimum of 50% *L. plantarum* as inoculant would probably make desirable ML silage.

#### Active Ingredients and Antioxidant Activity of Mulberry Leaves Silage

**FIGURE 1:**
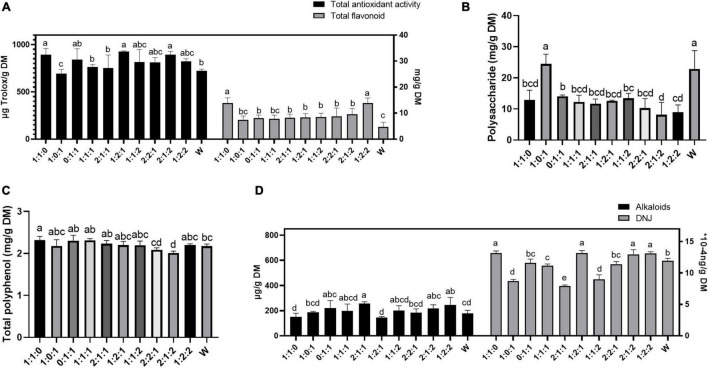
Total antioxidant activity and total flavonoid **(A)**, polysaccharide **(B)**, total polyphenol **(C)**, and alkaloids and DNJ **(D)** of mulberry leaves ensiled with different inoculant ratios in **trial 1**. Different letters in the same index among groups differed (*P* < 0.05).

As shown in Figure 1, there was a tendency to increase the contents of flavonoids, polyphenols, alkaloids, DNJ, and total antioxidant capacity in inoculated ML silage (1:1:0) compared with W, whereas polysaccharide content was decreased and in full accord with WSC. During the microbial fermentation, in addition to synthesizing enzymes to metabolize the ingredients of the substrate, the microorganisms also secreted some active ingredients to endow the substrate special functions, such as antioxidant properties. In addition, variation in the amount of every active ingredient depended on substrate source and inoculated bacteria (Liu et al., 2018; Li et al., 2021).

Overall, the load of both ≥ 50% of *L. plantarum* and the absence of *B. subtilis* as inoculants was sufficient to achieve successful fermentation of ML, followed by a speculation that < 1:1:0 (e.g., 0:1:0) would be a better choice than 1:1:0 to ferment ML. As reported, the outstanding inoculant ratio of 1:1:0 in trial 1 would be applied in trial 2 to test the fermentation conditions of ML silage. Synchronously, DM loss, pH, ammonia-N and amino acid contents, total antioxidant activity, and total flavonoids content were taken as meaningful indicators of evaluating ML silage quality in trial 2 and trial 3.

### Ensiling Characteristics of Mulberry Leaves Ensiled With Different Conditions (Trial 2)

**FIGURE 2:**
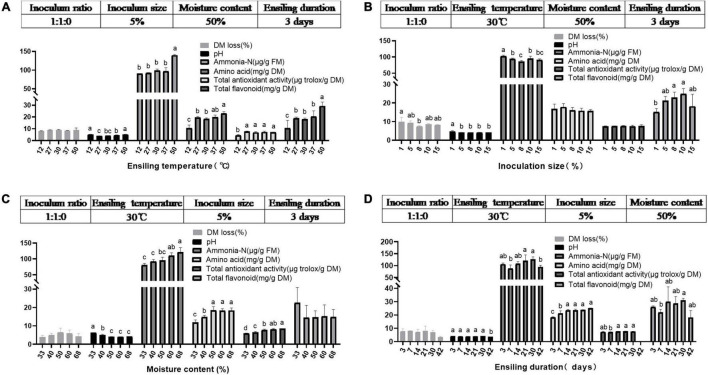
Characteristics of ML silage under various ensiling temperatures **(A)**, various inoculum sizes **(B)**, various moisture levels **(C)**, and various ensiling duration **(D)** using the single-factor experimental method in **trial 2**. Different letters in the same index among groups differed (*P* < 0.05).

It is generally assumed that ensiling temperature is a crucial factor impacting silage quality (Wang et al., 2019c). Various temperatures (12, 27, 30, 37, and 50°C) were adopted to prepare ML silage using the inoculant ratio 1:1:0. As expected (Figure 2A and Table 4), at low temperature (12°C), the pH of ML was higher, and the contents of amino acids and total flavonoids, and total antioxidant activity dramatically decreased. While fungi increased sharply, which could enhance fiber digestibility at a suitable amount in the rumen (Zhou et al., 2019) yet accompanied with nutrient loss of ML silage. And the obvious increase in undesirable Enterobacter caused putrefaction of ML silage (as observed also at 37°C). Wang et al. (2019c) reported that LAB inoculants and a relatively low ensiling temperature (15°C) could effectively improve the quality of silage of *Moringa oleifera* leaves, but the reason underlying this observation remains to be elucidated. Comparatively, a higher pH and ammonia-N content could be found in ML silage produced at higher ensiling temperature (50°C). Moreover, although the load of *Lactobacillus* was higher at 50°C, higher temperatures give bacilli an advantage over other LAB species in terms of competition for the available carbohydrates (Pahlow et al., 2003. Evidently, ensiling temperatures between 27 and 30°C could improve the quality of ML silage by ensuring low pH, lower ammonia content, and decreased number of *Enterobacter*.

**TABLE 4:** Microbial load determined by qPCR (log_10_CFU/g FM) of ML silage produced under different ensiling temperatures and duration in **trial 2**.

	*Lactobacillus*	Fungi	*Enterobacter*
**Fermentation temperature (°C)**			
12	6.25^a^	8.48^a^	5.31^a^
27	4.35^bc^	6.83^b^	4.37^ab^
30	4.09^c^	6.61^b^	4.44^ab^
37	5.19^b^	7.25^b^	5.29^a^
50	6.69^a^	7.28^b^	3.61^b^
SEM	0.293	0.193	0.219
*P*-value	0.000	0.002	0.036
**Ensiling duration (days)**			
3	5.01^a^	6.61^a^	5.13
7	5.03^a^	6.76^a^	5.14
14	4.95^a^	6.48^ab^	4.82
21	5.02^a^	6.48^ab^	4.55
30	4.80^a^	6.38^ab^	4.55
42	4.32^b^	6.10^b^	4.41
SEM	0.070	0.067	0.121
*P*-value	0.002	0.063	0.376

*Different letters in the same column (a–c) differed (P < 0.05).*

In addition, the effects of different inoculum sizes (1, 5, 8, 10, and 15%) and moisture contents (33, 40, 50, 60, and 67%) on the quality of ML silage prepared with the inoculant ratio 1:1:0 were evaluated. As shown in Figure 2B, inoculum sizes had less effect on the quality of ML silage; a low inoculum size (1%) was not conducive to pH decrease and/or flavonoids production (*P* < 0.05). Muck (2010) reported that less than 1% of the inoculant relative to the epiphytic population produced no significant changes in fermentation. Moreover, at low moisture content of ML (33 and 40%), ensiling did not result in a decrease in pH and/or increase in amino acids content and total antioxidant activity (*P* < 0.05; Figure 2C). It is well known that high pH silage is undesirable, since at these conditions the activity of *Clostridium* is maintained (Emerstorfer et al., 2011), thus leading to an increase in butyric acid production (Szymanowska-Powalowska et al., 2014). Increased levels of butyric acid in silage (> 5 g/kg DM) can lead to decreased feed intake and health issues of livestock (Muck, 2010).

Duration of ensiling is another key factor for silage quality (Zeng et al., 2020). Therefore, the effect of different ensiling duration (3, 7, 14, 21, 30, and 42 days) on ML silage quality prepared with inoculant ratio 1:1:0 was evaluated. Dynamic changes in ensiling parameters and bacterial load are presented in Figure 2D and Table 4, respectively. Overall, with prolonged ensiling time, bacterial load decreased, with the lowest load of *Lactobacillus* occurring on day 42. Simultaneously, the content of amino acids in ML increased over time, peaking on day 14.

Collectively, the most promising parameters for effective ML ensiling at the initial stage in this research included the following: temperature, 27–30°C; inoculum size, 5–15%; moisture content, 50–67%, and 14–30 days of ensiling.

### Ensiling Characteristics and Microbial Diversity of Mulberry Leaves Silage in Four Certain Conditions (Trial 3)

**FIGURE 3:**
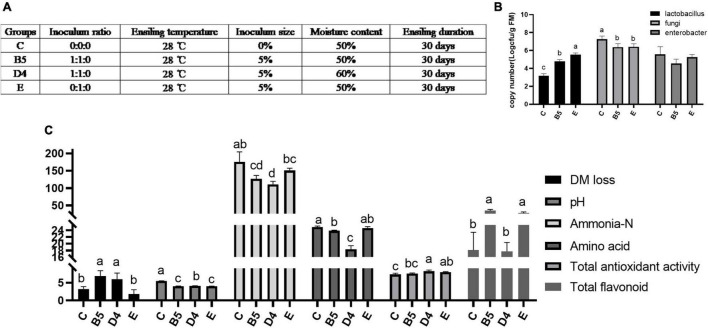
Ensiling characteristics **(C)** and microbial load by real-time fluorescence quantitative PCR **(B)** of mulberry leaves silage in **trial 3** with experimental design **(A)**. Different letters in the same index among groups differed (*P* < 0.05).

Trial 3 was set as control (C) and treatments (B5, D4, E) based on a synthesis of the preceding two experiments (Figure 3A). It aimed at creating a comprehensive road map to ferment ML practically and making sense of the differences and roles of the microbial community in inoculated ML silage. Changes in ensiling profiles and microbial population of ML silage are shown in Figures 3B,C. pH value is considered an important indicator reflecting ensiling quality. In this study, ML silage produced under B5 and E conditions had the lowest pH value (*P* < 0.05), possibly due to the heavier presence of species *Lactobacillus* quantified by PCR (*P* < 0.05), which produced more lactic acid to lower pH value in ML in accordance with Gallegos et al. (2017). High moisture content is presumably responsible to lower the acid concentration relative to the activities of LAB and yeasts (Muck, 2010), thus leading to a higher pH observed in D4 than B5 yet with the same *Lactobacillus* load. In addition, in ML produced under B5 and D4 conditions, the levels of ammonia, always indicative of protein breakdown, were remarkably reduced compared with the control, which can be attributed to a decrease in the load of *Enterobacter* (B5) as an ammonia-forming species. Notably, ML moisture content was closely related to ensiling quality, since at higher ML water content (B5 compared with D4) a greater decrease in amino acids and flavonoids contents was observed. Compared with B5, ML produced under E condition had a remarkable decrease in DM loss (*P* < 0.05) and an increase in the load of beneficial *Lactobacillus* (*P* < 0.05). But subsequent work could elucidate exactly how changes could lead to a substantial increase in flavonoids (*P* < 0.05) in B5 and E compared with C and D4.

**FIGURE 4:**
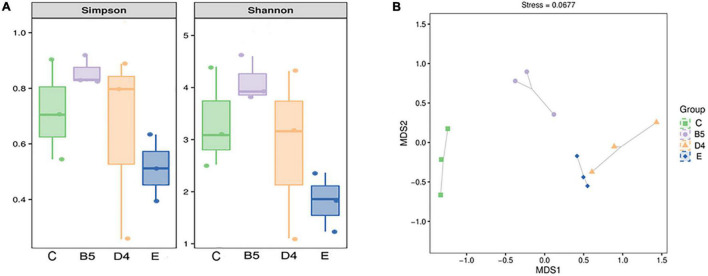
Simpson and Shannon indices **(A)** and Bray-Curtis **(B)** based on non-metric multidimensional scaling in **trial 3**.

**FIGURE 5:**
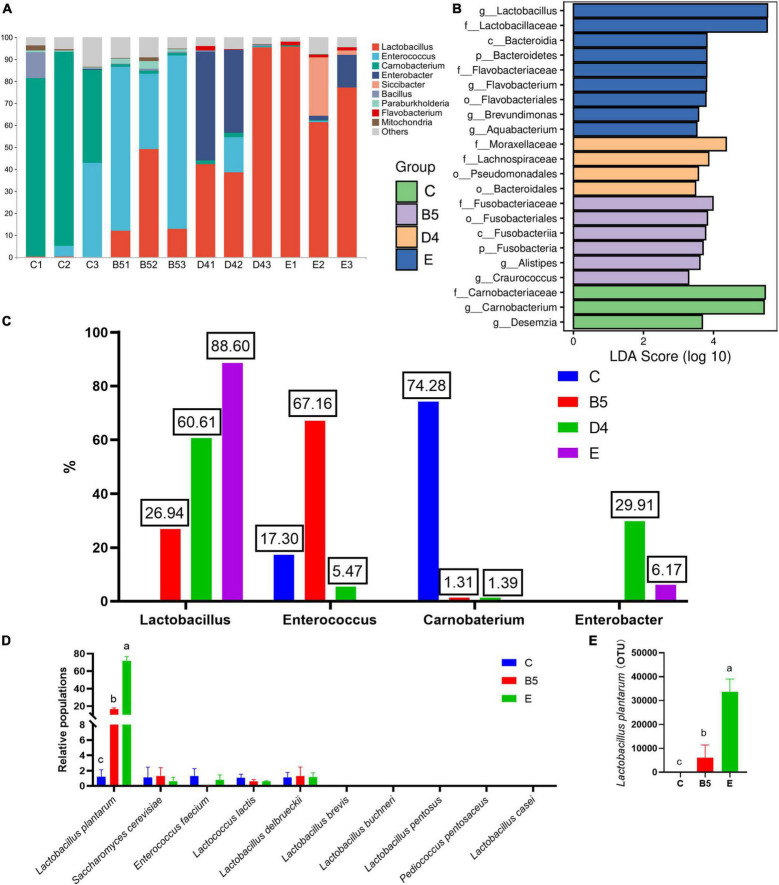
Relative abundances of microbial communities at the genus level **(A)**, comparison of microbial variations **(B)**, percentages of main bacteria in **A (C)**, populations of individual species of lactic acid bacteria by relative quantification PCR **(D)** and 16 s sequencing **(E)** among groups of **trial 3**.

Shannon and Simpson indices are commonly used to reflect the α-diversity of a bacterial community. These indices were increased in ML silage prepared under conditions (B5 and D4) and decreased in E, compared with C (Figure 4A). It hinted that bacterial diversity in ML silages was associated with inoculants. *Lactobacillus* (100% added) helped dominate a rapid fermentation. At the same time, contributing to lower microbial diversity in ML silage with abundant *Lactobacillus* (Figure 5A) after fermentation. Similar results have been reported previously (Wang et al., 2019b,c), indicating that a less diverse bacterial community is established in silages where *Lactobacillus* was used as an inoculant. Dominant lactobacillus could create an acidic environment to limit the growth of other microbes (Mendez-Garcia et al., 2015), thus to lower the diversity of the bacterial community (Ogunade et al., 2016).

As shown in Figure 4B, the result of β-diversity analysis based on non-metric multidimensional scaling clearly reflected the between-group variance of the microbial community. ML silages produced under conditions B5 and E groups treated with different inoculants were separated from the C group. Interestingly, a distinct separation was observed among bacterial communities in the same inoculated ML silages (B5 compared to D4). This observation indicated that not only the inoculant but also the moisture content had a remarkable effect on the microbial composition of silage (Wang et al., 2019c). The variation of microbial community composition might explain the difference in silage quality (Ni et al., 2017). But the inoculated silages of whole crop maize (Parvin et al., 2010) showed less or no change in the bacterial community composition.

Most abundant bacterial communities in ML silage are shown in Figure 5A. Overall, Firmicutes was the most abundant phylum in all treatments, followed by Cyanobacteria and Proteobacteria. Similarly, Wang et al. (2019b) and Liu et al. (2019) reported that the dominant phyla were Firmicutes and Proteobacteria in ML and barley silages with or without inoculants. The higher abundance of Firmicutes and Proteobacteria in ML silage might be attributed to low pH and generation of anaerobic conditions toward the end of ensiling (Wang et al., 2019b). Dominant genera in each treatment were shown in Figure 5C (*Lactobacillus*, 88.60% in group E; *Lactobacillus*, 60.61 and *Enterobacter*, 29.91 in group D4; *Lactobacillus*, 26.94% and *Enterococcus*, 67.16% in group B5; *Enterococcus*, 17.30% and *Carnobaterium*, 74.28% in group C). Moreover, the most differentially abundant genera were *Lactobacillus* (E), *Enterobacter* (D4), *Enterococcus* (B5), and *Carnobacterium* (C) (Figures 5B,C). There was one further point that inoculated *S. cerevisiae* do not persist well, but inoculant of *L. plantarum* in treatments B5 and E provided higher quality silage, also with persistence in well-preserved silage (Figures 5D,E). It appears that inoculants fulfill their role as starter cultures that strongly influence the earliest stage of silage fermentation, after which their persistence may or may not be necessary (Stevenson et al., 2006). The well-persisted *L. plantarum* stands out in inoculated strains because of its domination, reliability, ability to use different sugars, easy adaptation to various media, and probiotic properties (Kamiloglu et al., 2020). The lower population of *S. cerevisiae* after fermentation might be present only in the earlier ensiling stage, while environmental pH was relatively high before high activities of lactobacillus, to contribute to sensory qualities of silage (Suranska et al., 2016) or to create anoxic environment for *L. plantarum* productivity or to alter the diversity of community microorganisms. Also, the populations of *Enterococcus faecium*, *Lactococcus lactis*, and *Lactobacillus delbrueckii* were determined at lower insignificant levels comparable with *S. cerevisiae* between treatments, and there were undetectable *Lactobacillus brevis*, *L. buchneri*, *Lactobacillus pentosus*, *Pediococcus pentosaceus*, and *Lactobacillus casei* (Figure 5D). The result dropped a hint that *E. faecium*, *L. lactis*, and *L. delbrueckii* had been working during the production of ML silage.

The genera *Lactobacillus*, *Enterococcus*, *Lactococcus*, *Weissella*, and *Pediococcus*, as common lactic acid-producing bacteria, participate positively in the evolution of LAB population during ensiling and have been widely used to improve silage quality (Chen et al., 2012; Yang et al., 2016; Ni et al., 2018). Generally, lactic acid-producing cocci (i.e., *Weissella*, *Leuconostoc*, *Pediococcus*, *Lactococcus*, and *Enterococcus*) initiate lactic fermentation at the early stages of ensiling, whereas lactic acid-producing bacilli (*Lactobacillus*) grow vigorously as pH decreases, hence becoming the dominant species at late stages of ensiling (Cai et al., 1998; Zhou et al., 2016; Liu et al., 2019). Moreover, it is known that homofermentative LAB can decrease the pH of silage by producing lactic acid (Oliveira et al., 2017), whereas heterofermentative LAB produced high concentrations of acetic acid, 1,2-propanediol, and other products to increase the stability of silage against deterioration by undesirable microorganisms when exposed to air (Guo et al., 2018). In this regard, the higher abundance of *Lactobacillus* in ML silage in group E, as well as after ensiling, revealed that the use of *Lactobacillus* as an inoculant was conducive to high-quality ensiling and may partly explain changes in DM loss, pH, amino acid content, and lower bacterial diversity. Cai et al. (1998) and Ogunade et al. (2016) reported that *Enterococcus* plays a pivotal role in accelerating LA fermentation and in building an anaerobic acidic environment for the development of *Lactobacillus* and to survive only at the early stages of ensiling due to its sensitivity to acid.

Interestingly, a higher abundance of *Enterococcus* was observed in ML silages with combined inoculum (Figure 5A), which might be explained by the different bacteria of *Alistipes* and *Craurococcus* (Figure 5B). The presence of *Alistipes* was significantly positively correlated with SCFAs, which are the end products of amino acid fermentation (David et al., 2013). *Craurococcus* was significantly positively correlated with total nitrogen content and negatively correlated with pH value (Rodríguez-Berbel et al., 2020); this species can degrade nutrients to produce ammonia and mitigate acidic pH, leading to a slow decrease in pH of ML silage and a higher microbial diversity except for proliferation of *Enterococcus*. This finding was consistent with increased DM loss and decreased ammonia content (B5). Moreover, the low pH value at the end of ensiling was a result of the increased abundance of *Lactobacillus*.

As the main competitors of LAB for nutrients in silage, enterobacteria produce ammonia and convert lactic acid to acetic acid alongside gas production (Muck, 2010), which reduces the energy and nutritive value of ML silage. Accordingly, increased DM loss of ML silage might be caused by the higher abundance of *Enterobacter* in group D4 due to higher moisture content, which leads to high pH and a more diverse bacterial community. Wang et al. (2019b) found that a higher abundance of *Enterobacter* could lead to relatively high levels of acetic acid and ammonia. However, pH in ML silage of group D4 was lower, which might be related to an increased proportion of *Lactobacillus*.

*Carnobacterium* is a Gram-positive LAB species that can metabolize arginine and various carbohydrates, frequently predominating in a range of foods, including fish, meat, and certain dairy products (Leisner et al., 2007; Mills et al., 2018). Notably, *Carnobacterium* has only been recently reported, and its roles were less described in silage. He et al. (2020a) discovered that the abundance of *Carnobacterium* was increased in control ML silage, which was consistent with the findings presented in this study. In addition, *Carnobacterium* has been found to be abundant in alfalfa silage obtained without additives and in fresh oat according to Wang et al. (2020) and Bai et al. (2020). Therefore, it could be interpreted as the change in silage pH, DM loss, and ammonia-N in group C benefited the growth of *Carnobaterium*, which was cultured to a final pH of 5.4 with an optimal growth at alkaline pH (Afzal et al., 2013) with weak acid-producing and acid-tolerance abilities; led to an increase in pH and the abundance of spoilage genera such as *Clostridium* (He et al., 2020b); or caused silage spoilage and off-odors *via* the production of spoiled metabolites (Leisner et al., 1995). Collectively, variations in microbial community composition might explain differences in silage quality, and the analysis of bacterial abundance suggested that optimized conditions for ML ensiling comprised the use of *Lactobacillus* or the combined use of *Lactobacillus* and yeast as inoculants.

**FIGURE 6:**
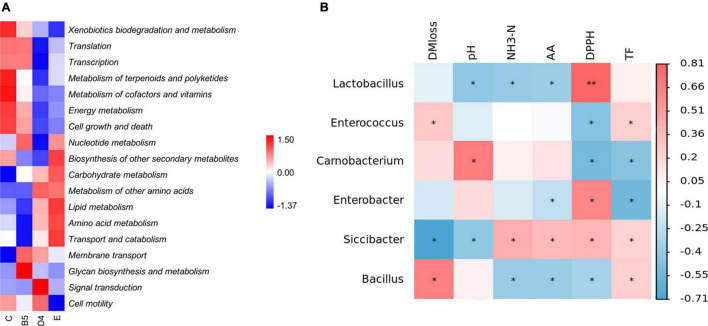
Heatmap of 16S rRNA gene predicted functional profiles **(A)** and Pearson’s correlation analysis of the bacterial community composition and ensiling parameters **(B)** in **trial 3**. *P < 0.05; **P < 0.01.

The 16S rRNA gene-predicted functional profiles are shown in Figure 6A. Metabolism of carbohydrates, amino acids, and lipids was slightly increased in ML silage of group E along with a higher abundance of *Lactobacillus*, which contrasted with the findings obtained by Wang et al. (2019a), who found that a higher abundance of *Lactobacillus* would prompt a reduction in the metabolism of nitrogen, arginine, proline, glycine, serine, and threonine in alfalfa and stylo silage mixed with *M. oleifera* leaves. This discrepancy and decrease in DM loss previously described were related to nutrient and secondary metabolite metabolism. Consistently, the biosynthesis of other secondary metabolites was increased (E), leading to increased production of total flavonoids and improved antioxidant activity as aforementioned. The improved glycan metabolism in group B5 might be related to membrane transport, transcription, and translation, as glycans by themselves might act as signaling molecules internal to a species (Varki, 2017). Simultaneously, the higher metabolism of energy, cofactors and vitamins, terpenoids, and polyketides in group C resulting in more DM loss was likely due to abundant Carnobacteria.

To explore the relationships among ensiling properties and the roles of main bacteria acting on the fermentation process of ML, a heatmap of Pearson’s correlation analysis of the bacterial community at the genus level (Figure 6B) and fermentation parameters was employed. Usually, metabolites are positively correlated with profitable microorganisms and negatively correlated with undesirable bacteria during ensiling based on the interaction between chemicals and microbial activity (Xu et al., 2019). *Siccibacter*, prior belonging to the genus *Cronobacter*, was reclassified as a new genus in 2014 and comprises non-pathogenic strains, isolated mainly from fruit powders, spices, herbs, and infant formula (Svobodova et al., 2017). According to the correlation analysis in this study, it was speculated that *Siccibacter* was probably linked to decreased DM loss, and similar to *Lactobacillus*, it might have the powerful ability to lower pH in ML silage than *Carnobacterium*, in spite of its small population in entire microflora. As reported, *Siccibacter turicensis* played beneficial roles in the cellulases, such as the endoglucanase (Dhakal et al., 2020), and thus, *Siccibacter* could potentially be exploited as a probiotic microbe for ML silage. For nutrient substances, *Enterobacter* correlated with decreased amino acid and total flavonoids contents. Furthermore, it was also noted that the presence of *Lactobacillus* could lead to reduced ammonia-N and improved antioxidant activity in ML silage. Besides, it was interesting to find that the relative abundance of bacteria was dissociated from their activity during ensiling.

In summary, successfully fermented ML silage should be used as livestock feed under condition E with the lowest DM loss and pH, followed by B5. Analysis of the microbial community revealed altered fermentation patterns between B5 fermented exclusively with *L. plantarum* (0:1:0) and E with combined *S. cerevisiae* and *L. plantarum* (1:1:0). Additionally, Group E, dominated by *Lactobacillus* (88.60%), exhibited less bacterial diversity and higher levels of nutrient and other secondary metabolite metabolism but depressed glycan metabolism than B5 with abundant *Enterococcus* (67.16%) and *Lactobacillus* (26.94%). In addition, the inoculant of *L. plantarum*, not *S. cerevisiae*, persists well in the well-preserved silage, which was a hint of a crucial role of *L. plantarum* acting in ML fermentation.

## Conclusion

In conclusion, successful ensiling of ML requires inoculants and appropriate ensiling conditions, and inoculants could alter abundant bacteria, thus affecting certain metabolism to obtain glycan (B5) or small abundant secondary metabolite (E). However, the molecular mechanism of nutrient and secondary metabolite metabolism in ML silage after inoculation should be studied more deeply in the future. In this study, one further important point was the good persistence of *L. plantarum*, but not *S. cerevisiae*, in the well-preserved ML silage.

## Data Availability Statement

The datasets presented in this study can be found in online repositories. The names of the repository/repositories and accession number(s) can be found in the article/Supplementary Material.

## Author Contributions

XC and YY contributed to the conception and design of the study. XC performed the statistical analysis and wrote the first draft of the manuscript. YY, MZ, FJ, CS, and YQ revised the manuscript. TG, ZL, YH, HW, SL, and LB helped with the experimental sections. CS and YQ provided financial support for the manuscript. All authors contributed to manuscript revision, and read and approved the submitted version.

## Conflict of Interest

The authors declare that the research was conducted in the absence of any commercial or financial relationships that could be construed as a potential conflict of interest.

## Publisher’s Note

All claims expressed in this article are solely those of the authors and do not necessarily represent those of their affiliated organizations, or those of the publisher, the editors and the reviewers. Any product that may be evaluated in this article, or claim that may be made by its manufacturer, is not guaranteed or endorsed by the publisher.
