# Supplementary figures and images for: Optimized Ensiling Conditions and Microbial Community in Mulberry Leaves Silage With Inoculants
Source: Front Microbiol. 2022 Jun 2;13:813363. doi: 10.3389/fmicb.2022.813363 (PMC9201477; doi:10.3389/fmicb.2022.813363)

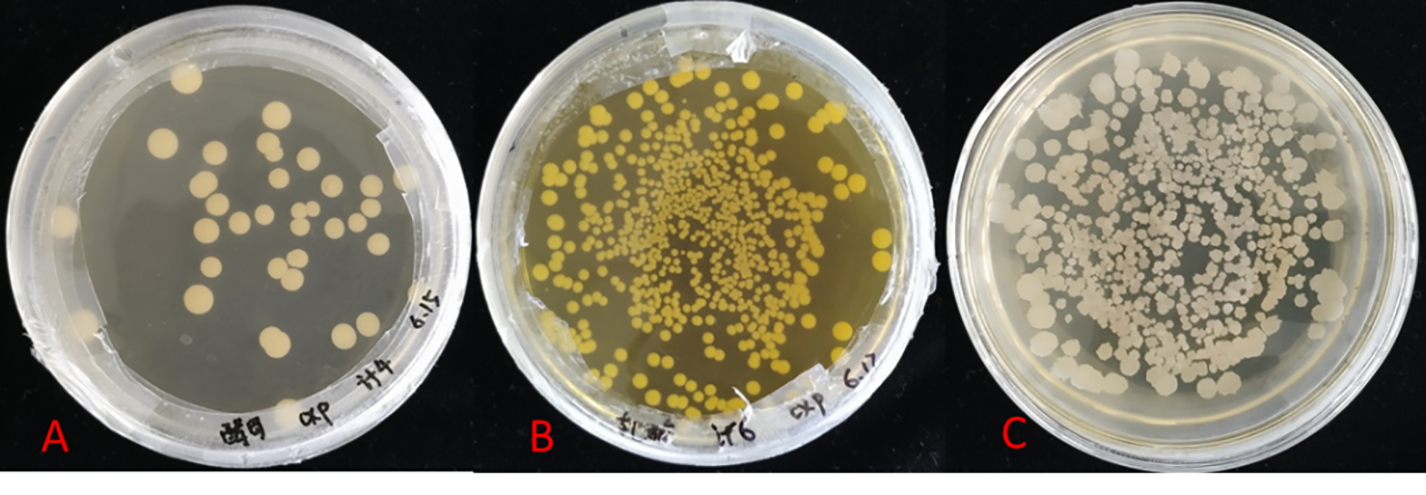

Supplement: Supplementary file 1 [file Image_1.TIF]

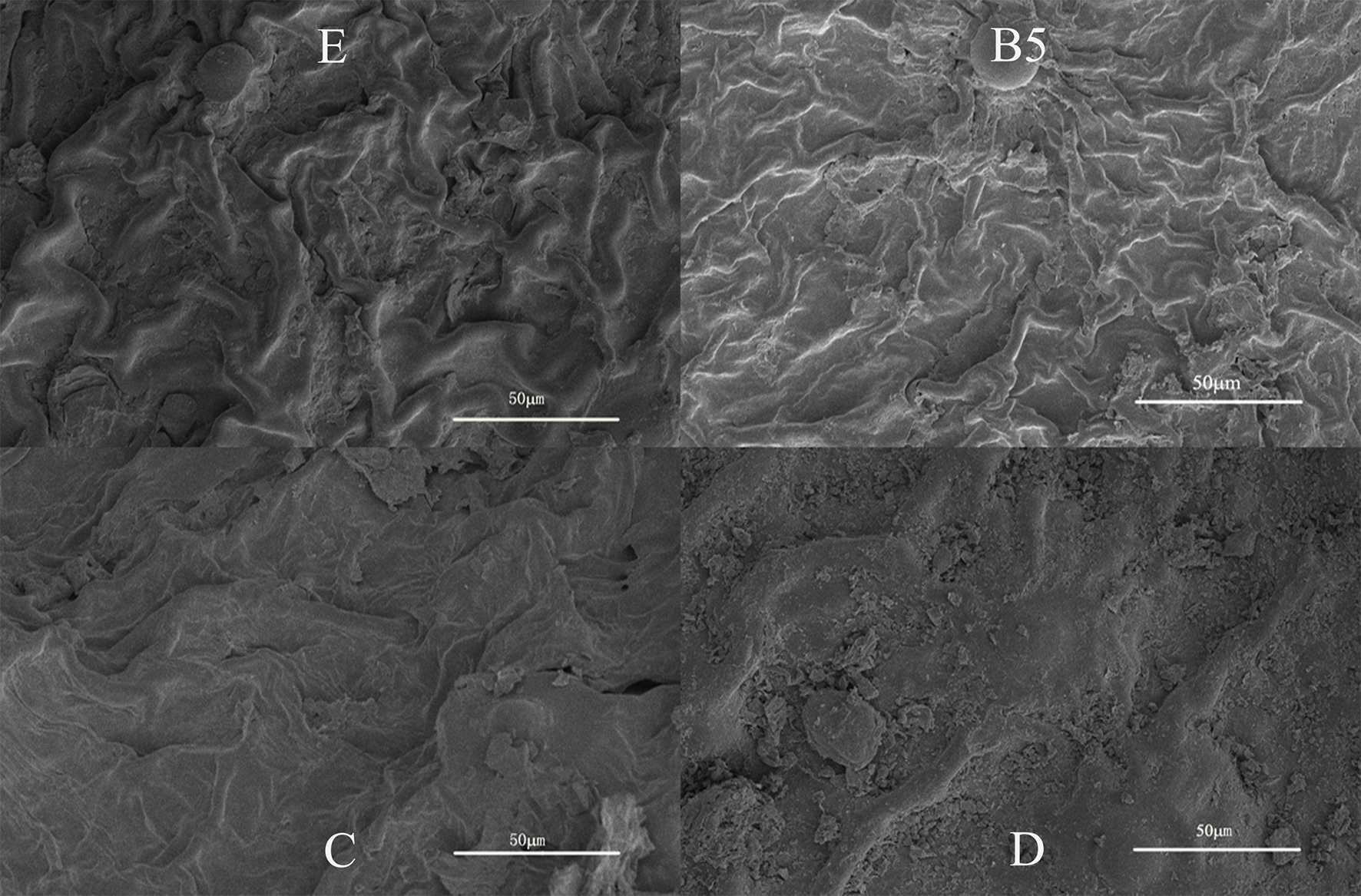

Supplement: Supplementary file 2 [file Image_2.TIF]

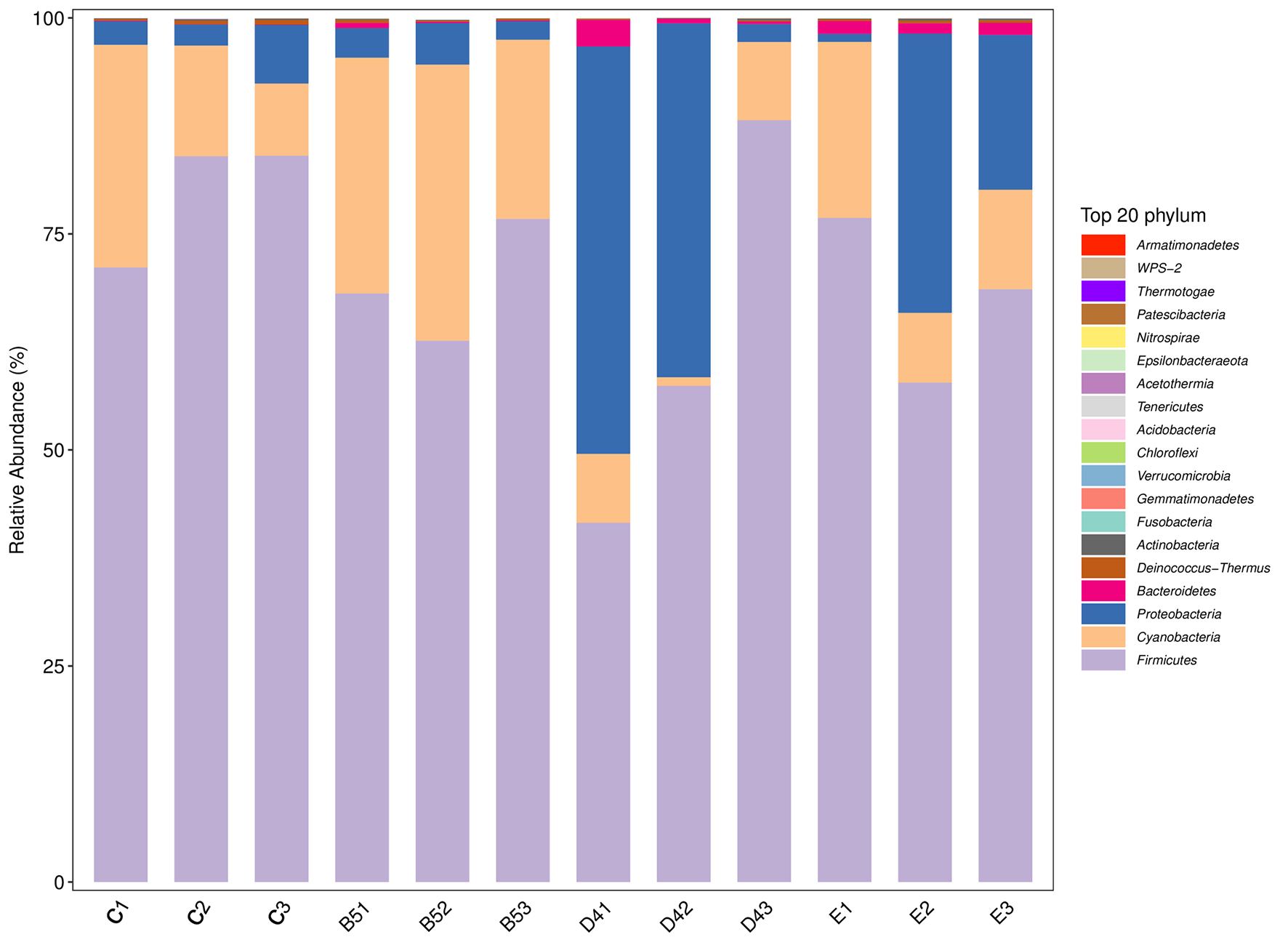

Supplement: Supplementary file 3 [file Image_3.TIF]

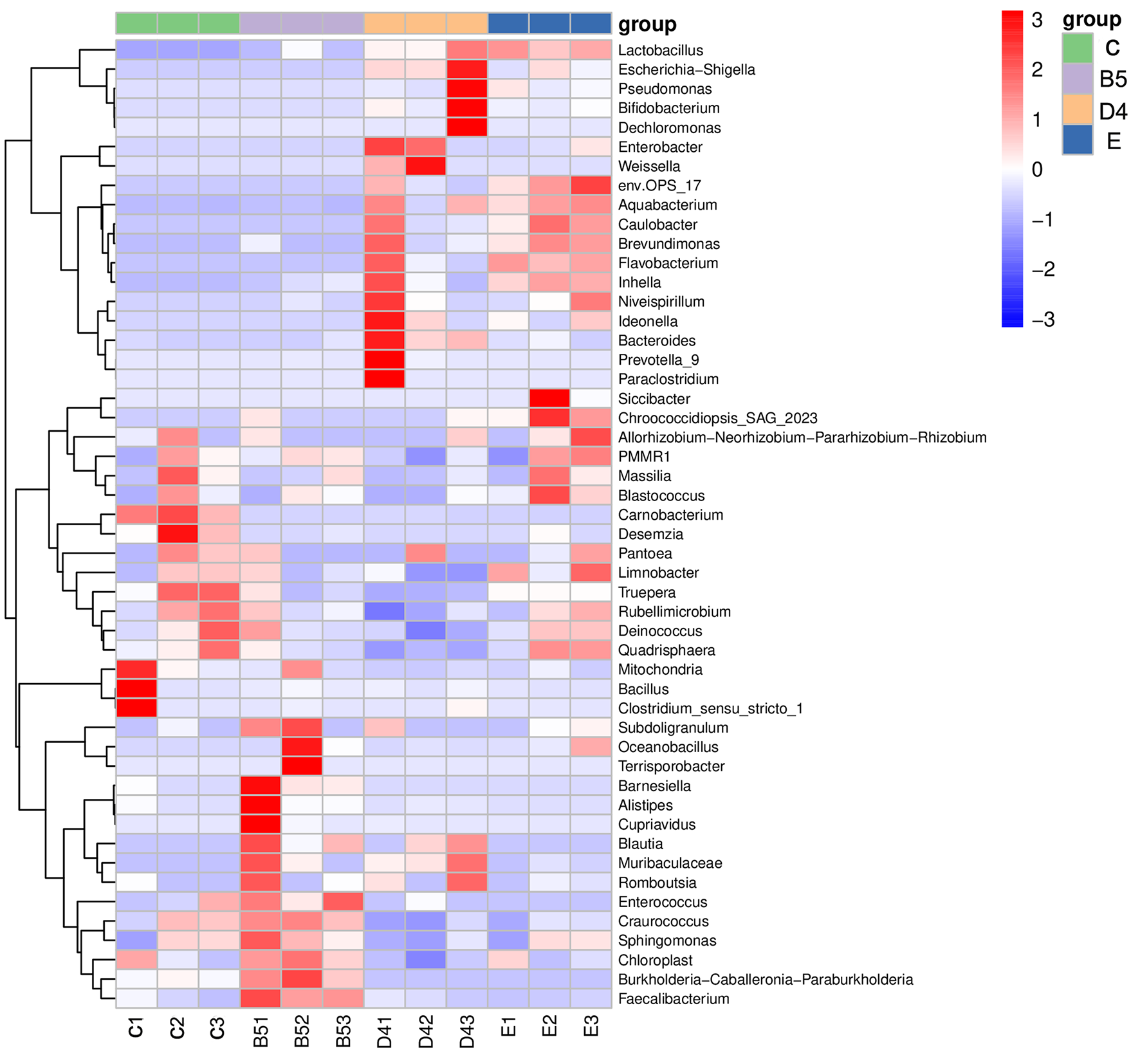

Supplement: Supplementary file 4 [file Image_4.TIF]
