# Supplementary material for: Optimized Ensiling Conditions and Microbial Community in Mulberry Leaves Silage With Inoculants
Source: Front Microbiol. 2022 Jun 2;13:813363. doi: 10.3389/fmicb.2022.813363 (PMC9201477; doi:10.3389/fmicb.2022.813363)
Supplement: Supplementary file 5 [file Table_1.DOCX]

Supplementary Material

# Supplementary Tables

**Table S1** Primers designed for quantitative PCR of *Lactobacillus*, fungi and *Enterobacter* in mulberry leaves silage.

| PCR target (gene) | Primer sequence (5’–3’) | Amplicon size (bp) | References |
| --- | --- | --- | --- |
| *Lactobacillus*  (16S) | UF-lac: TTTAYGCGGAACAYYTRGGKGT  UR-lac: CCAAACATCACVCCRACTT | 450 | [1] |
| Fungi | F: TGACTCAACACGGGGAAACT  R: CCAACTAAGAACGGCCATGC | 105 | [2] |
| *Enterobacter*  (16S) | F: ATCAGATGTGCCCAG ATGG  R: CCGTGTCTCAGTTCCAGTG | 110 | [3] |

1. Peng, K., Jin, L., Niu, Y.D., Huang, Q., McAllister, T.A., Yang, H.E., Denise, H., Xu, Z., Acharya, S., Wang, S., Wang, Y., Elliot, M.A. 2018. Condensed Tannins Affect Bacterial and Fungal Microbiomes and Mycotoxin Production during Ensiling and upon Aerobic Exposure. Appl Environ Microbiol, 84(5), 1-48.
2. Zhou, R., Wu, J., Zhang, L., Liu, L., Casper, D.P., Jiao, T., Liu, T., Wang, J., Lang, X., Song, S., Gong, X. 2019. Effects of oregano essential oil on the ruminal pH and microbial population of sheep. PLoS One, 14(5), 1-14.
3. Patel, C.B., Shanker, R., Gupta, V.K., Upadhyay, R.S. 2016. Q-PCR Based Culture-Independent Enumeration and Detection of Enterobacter: An Emerging Environmental Human Pathogen in Riverine Systems and Potable Water. Front Microbiol, 7, 1-10.

**Table S2** Statistical sequencing amount per sample

| **SampleID** | **Input** | **Filtered** | **Denoised** | **Merged** | **Non-chimeric** | **Non-singleton** |
| --- | --- | --- | --- | --- | --- | --- |
| C1 | 93582 | 79845 | 79181 | 78162 | 68903 | 68798 |
| C2 | 92561 | 79742 | 79118 | 78154 | 70665 | 70603 |
| C3 | 90200 | 77206 | 76492 | 75615 | 59883 | 59766 |
| B51 | 90843 | 78537 | 77821 | 76829 | 66081 | 65959 |
| B52 | 93066 | 79630 | 78964 | 77863 | 64798 | 64671 |
| B53 | 89370 | 76701 | 76163 | 75444 | 62921 | 62849 |
| D41 | 80461 | 73129 | 72558 | 71976 | 65451 | 65355 |
| D42 | 74277 | 67869 | 67299 | 66675 | 54011 | 53928 |
| D43 | 75504 | 69094 | 68709 | 68349 | 67799 | 67740 |
| E1 | 101071 | 92471 | 91909 | 91369 | 87568 | 87510 |
| E2 | 98782 | 88410 | 87702 | 86821 | 77025 | 76906 |
| E3 | 105326 | 95847 | 95125 | 94363 | 86526 | 86396 |

**Table S3** Pearson correlation coefficient matrix of ensiling characteristics, active ingredients, and antioxidant activity of ML silage with the addition of different inoculant combinations

|  | DM_loss_ | Ammonia-N | pH | LA | AA | LAB | CP | Amino acid | WSC | TF | P | TP | A | DNJ | DPPH |
| --- | --- | --- | --- | --- | --- | --- | --- | --- | --- | --- | --- | --- | --- | --- | --- |
| DM_loss_ | 1.000 |  |  |  |  |  |  |  |  |  |  |  |  |  |  |
| Ammonia-N | -0.260 | 1.000 |  |  |  |  |  |  |  |  |  |  |  |  |  |
| pH | -0.152 | 0.493** | 1.000 |  |  |  |  |  |  |  |  |  |  |  |  |
| LA | 0.209 | -0.523** | -0.248 | 1.000 |  |  |  |  |  |  |  |  |  |  |  |
| AA | 0.100 | -0.407* | -0.053 | 0.942** | 1.000 |  |  |  |  |  |  |  |  |  |  |
| LAB | 0.328 | -0.339 | -0.034 | 0.761** | 0.742** | 1.000 |  |  |  |  |  |  |  |  |  |
| CP | -0.002 | 0.218 | 0.663** | 0.230 | 0.295 | 0.253 | 1.000 |  |  |  |  |  |  |  |  |
| Amino acid | 0.306 | 0.202 | 0.346* | -0.163 | -0.092 | 0.049 | 0.183 | 1.000 |  |  |  |  |  |  |  |
| WSC | -0.175 | 0.615** | 0.882** | -0.341 | -0.079 | -0.108 | 0.479** | 0.364** | 1.000 |  |  |  |  |  |  |
| TF | 0.245 | -0.214 | -0.447** | 0.447** | 0.267 | 0.279 | -0.062 | -0.303 | -0.442** | 1.000 |  |  |  |  |  |
| P | -0.125 | 0.344* | 0.801** | -0.205 | 0.023 | 0.039 | 0.343 | 0.343 | 0.802** | -0.519** | 1.000 |  |  |  |  |
| TP | 0.201 | -0.284 | -0.070 | 0.408* | 0.413* | 0.474** | 0.031 | 0.209 | -0.056 | 0.164 | 0.030 | 1.000 |  |  |  |
| A | -0.210 | 0.053 | -0.106 | 0.036 | -0.011 | -0.267 | 0.107 | -0.142 | -0.170 | -0.151 | -0.189 | -0.151 | 1.000 |  |  |
| DNJ | 0.034 | -0.174 | -0.271 | 0.252 | 0.245 | 0.080 | -0.192 | -0.403* | -0.217 | 0.341 | -0.284 | -0.090 | -0.247 | 1.000 |  |
| DPPH | -0.033 | -0.317 | -0.494** | 0.125 | 0.009 | 0.097 | -0.418* | -0.393* | -0.494** | 0.444** | -0.402* | 0.112 | -0276 | 0.495** | 1.000 |

^1^ TF: Total flavonoid; P: Polysaccharide; TP: Total polyphenols; A: Alkaloids; DPPH: Total antioxidant activity.

^2^Values in each category indicate correlation coefficients; * or ** denote significant differences of correlation coefficients; Empty cells denote no significant differences of correlation coefficients

**Figure S1** Colony morphology of the three strains tested as inoculants in mulberry leaves silage. (A) *Saccharomyces cerevisiae*; (B) *Lactobacillus plantarum*; (C) *Bacillus subtilis*.

**Figure S2** Morphology of mulberry leaves silage by scanning electronic microscope (Magnification: 2000×); (D, dry ML).

**Figure S3** Relative abundance of microbial community in mulberry leaves silage at the phylum level.

**Figure S4** Heatmap of variations of microbial communities.
